# De novo transcriptome sequencing and analysis revealed the molecular basis of rapid fat accumulation by black soldier fly (*Hermetia illucens*, L.) for development of insectival biodiesel

**DOI:** 10.1186/s13068-019-1531-7

**Published:** 2019-08-09

**Authors:** Zhaolu Zhu, Kashif ur Rehman, Yongqiang Yu, Xiu Liu, Hui Wang, Jeffery K. Tomberlin, Sing-Hoi Sze, Minmin Cai, Jibin Zhang, Ziniu Yu, Jinshui Zheng, Longyu Zheng

**Affiliations:** 1 State Key Laboratory of Agricultural Microbiology, National Engineering Research Center of Microbial Pesticides, College of Life Science and Technology, Huazhong Agricultural University, Wuhan, People’s Republic of China; 2 Livestock and Dairy Development Department, Poultry Research Institute, Rawalpindi, Pakistan; 3 Department of Entomology, Texas A&M University, College Station, TX USA; 4 Department of Computer Science and Engineering, Department of Biochemistry & Biophysics, Texas A&M University, College Station, TX USA; 5 College of Informatics, Huazhong Agricultural University, Wuhan, China; 6 Insectplus, Apfelbaumstrasse 22, 8050 Zurich, Switzerland

**Keywords:** Black soldier fly, Fat accumulation mechanism, Transcriptome sequencing, Biodiesel, Biorefinery

## Abstract

**Background:**

Black soldier fly (BSF, *Hermetia illucens* L.) can efficiently degrade organic wastes and transform into a high fat containing insect biomass that could be used as feedstock for biodiesel production. Meanwhile, the molecular regulatory basis of fat accumulation by BSF is still unclear; it is necessary to identify vital genes and regulators that are involved in fat accumulation.

**Results:**

This study analyzed the dynamic state of fat content and fatty-acid composition of BSF larvae in eight different stages. The late prepupa stage exhibited the highest crude fat, with lauric acid being the main component. Therefore, to provide insight into this unexplained phenomenon, the molecular regulation of rapid fat accumulation by BSF larvae was investigated. The twelve developmental stages of BSF were selected for transcriptome analysis, including the eight stages used for investigation of fat content and fatty-acid composition. By Illumina sequencing, 218,295,450,000 nt were generated. Through assembly by Trinity, 70,475 unigenes were obtained with an average length of 1064 nt and an N50 of 1749 nt. The differentially expressed unigenes were identified by DESeq, with 9159 of them being up-regulated and 10,101 of them were down-regulated. The several putative genes that are involved in the formation of pyruvate, acetyl-CoA biosynthesis, acetyl-CoA transcription, fatty-acid biosynthesis, and triacylglycerol biosynthesis were identified. The four vital metabolic genes that are associated with fat accumulation were validated by quantitative real-time PCR (qRT-PCR). The molecular mechanism of fat accumulation in BSF was clarified in this investigation through the construction of a detailed fat accumulation model from our results.

**Conclusion:**

The study provides an unprecedented level of insight from transcriptome sequencing to reveal the crude fat accumulation mechanism in developing BSF. The finding holds considerable promise for insectival biodiesel production, and the fat content and fatty-acid composition can be altered by genetic engineering approaches in the future for the insect production industry.

**Electronic supplementary material:**

The online version of this article (10.1186/s13068-019-1531-7) contains supplementary material, which is available to authorized users.

## Background

The world’s available fuel resources are gradually depleting due to an increase in the energy demand; moreover, the use of fossil fuels is devastating our environment through greenhouse gases (GHG) emission and global warming. Therefore, the search for alternative and sustainable energy sources has attracted widespread attention in recent years to reduce the global environmental problem (GHG emission and global warming) and protect fossil energy resources. Currently, several alternatives are being studied and implement. The biodiesel is the most promising alternative fuel that provides environmental benefits, since there use leads to the decrease in the harmful emission of GHG and their affects [1]. Moreover, it was noted that biodiesel is renewable, non-toxic and biodegradable energy, with performance and characteristic that is very close to fossil diesel fuel [2]. As biodiesel is mainly produced from crop oil, the problems of limited feedstock, expensive production cost and competition with food resources have prevented its large-scale applications [3]; therefore, it is important to develop economical feedstock for biodiesel production.

The increase in the global human population resulted in a rise in the level of organic wastes products such as food waste, animal manure, and other agricultural wastes. Moreover, these organic wastes not only contribute to nutrient imbalances of soil results in soil quality deterioration; they also lead to water and air pollution [4]. Currently, the standard practices in the management of these wastes include disposal in landfills, combustion, or agricultural applications such as soil conditioner [5]. These organic wastes managemental practices have caused more critical environmental concerns, such as the creation of leachate and landfill gases, pest attraction, and water and air pollution [6, 7] considerably.

**Fig. 1:**
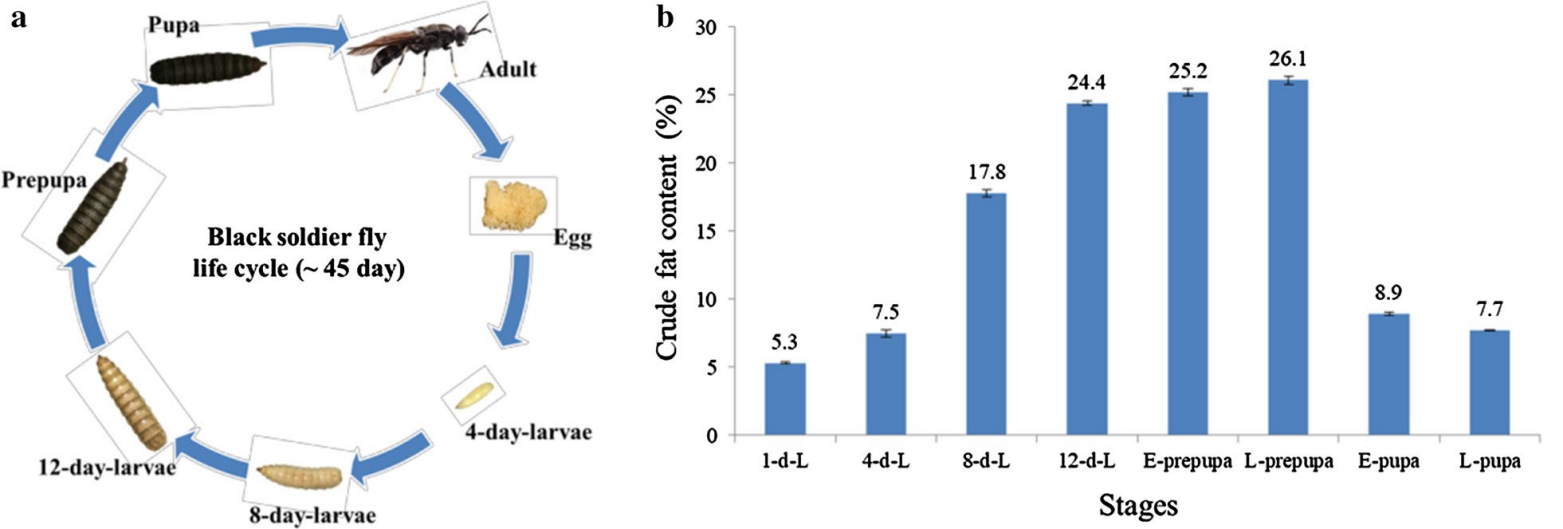
Graphical representation of the life cycle and fat content of black soldier fly. **a** Life cycle of black soldier fly. **b** Dynamic patterns of fat content in developing BSF. Bars indicate the standard error of the means (*n* = 3)

A more environmentally friendly and economically feasible approach is to take advantages of insects to convert organic wastes into larval biomass and biofertilizer through a process called biotransformation [6]. The black soldier fly larvae (BSFL) is a widely distributed insect can consume various organic material, from animal manure to vegetables and fruits [7]. The life cycle of BSF includes four phases: egg, larvae, pupa and adult stage (Fig. 1a). Since BSFL can transform various low-value organic wastes into protein and fat [8–10], and the fat content have 20–40% [11, 12]. So insect fat biomass can be used for biodiesel production that has many valuable properties, such as diesel compatibility, high energy content and cetane number, as was previously demonstrated by Zheng et al. [13, 14] and Rehman et al. [6, 15]. In our previous studies, fat extracted from BSFL is composed mainly of three fatty acids (FA), including lauric acid (C12:0), palmitic acid (C16:0) and oleic acid (C18:1) [6, 14]. Due to a large amount of saturated fatty acid and short carbon chain such as lauric acid (C12:0), the biodiesel produced by BSFL fat has high oxidative stability [6, 16]. The performance measurements for biodiesel produced by BSFL fat have shown fuel properties that are consistent with the international standard of biodiesel (EN14214) [15]. Therefore, the high-fat content and waste conversion efficiency of BSFL make it an ideal feedstock for biodiesel production [6].

The advances in sequencing technology have been considerable in recent years [17], such methods, especially those utilizing RNA sequencing (RNA-Seq), allow for examination of mechanisms responsible for metabolic variation [18]. These methods have been utilized on many insects, such as oriental fruit fly, mosquito *Culex pipiens*, and vinegar flies *Drosophila melanogaster*, to determine genes that encode enzymes associated with fatty acid (FA) and triacylglycerol (TAG) biosynthesis [19–22]. Although there are many researches on using BSFL fat for biodiesel production, whereas the molecular regulatory basis of fat accumulation by BSFL is still unclear. Thus, the transcriptomic analysis is essential to clarify the molecular regulatory mechanism of rapid fat accumulation by BSFL for the development of insectival biodiesel.

The present work studied the dynamic changes of fat content and FA composition in developing BSFL at eight stages, including one-day-old larvae (1-d-L), four-day-old larvae (4-d-L), eight-day-old larvae (8-d-L), twelve-day-old larvae (12-d-L), early prepupa (E-prepupa), late prepupa (L-prepupa), early pupa (E-pupa), and late pupa (L-pupa). Moreover, the four additional stages are also added for RNA-Seq, including early egg (E-egg), late egg (L-egg), female adult (F-adult), and male adult (M-adult), resulting in a total of twelve stages. The Illumina high-throughput sequencing was performed to obtain unigene annotations, screen differentially expressed unigenes by the DESeq method, and identify temporal expression patterns of putative genes involved in FA and TAG biosynthesis in BSFL. The designated routes of fat accumulation are based on transcriptome data from Illumina sequencing and temporal expression analysis. To reveal the molecular regulatory mechanism of rapid fat accumulation, the FA and TAG biosynthesis pathways in BSF were constructed to form a fat accumulation model that includes detailed information on when and how important genes are expressed and the relationships between its different components. Experimental validation of four vital genes was performed through quantitative real-time PCR (qRT-PCR). The results will provide a useful resource for functional genomics and enrich the public database. These resources will provide a foundation to increase fat accumulation and alter the FA composition of BSFL in the future.

## Results

### Dynamic patterns of fat content and FA compositions in developing BSF

To investigate the dynamic patterns of fat accumulation in developing BSFL, we analyzed the crude fat (CF) content in eight different stages (1-d-L, 4-d-L, 8-d-L, 12-d-L, E-prepupa, L-prepupa, E-pupa, and L-pupa) (Fig. 1b). It was noted that the CF was lower in BSFL during early stages (1-d-L to 4-d-L) and late stages (E-pupa to L-pupa), whereas higher fat content was recorded at mature larvae stage (12-d-L) and prepupa stages (E-prepupa to L-prepupa). A remarkable increase of CF was recorded at 4-d-L to 8-d-L from 7.5% to 17.8%, with the highest CF (26.1%) at E-prepupa stage. However, a sharp decline (17.2%) in CF was noted at E-pupa to L-pupa after the E-prepupa stage (26.1%).

**Fig. 2:**
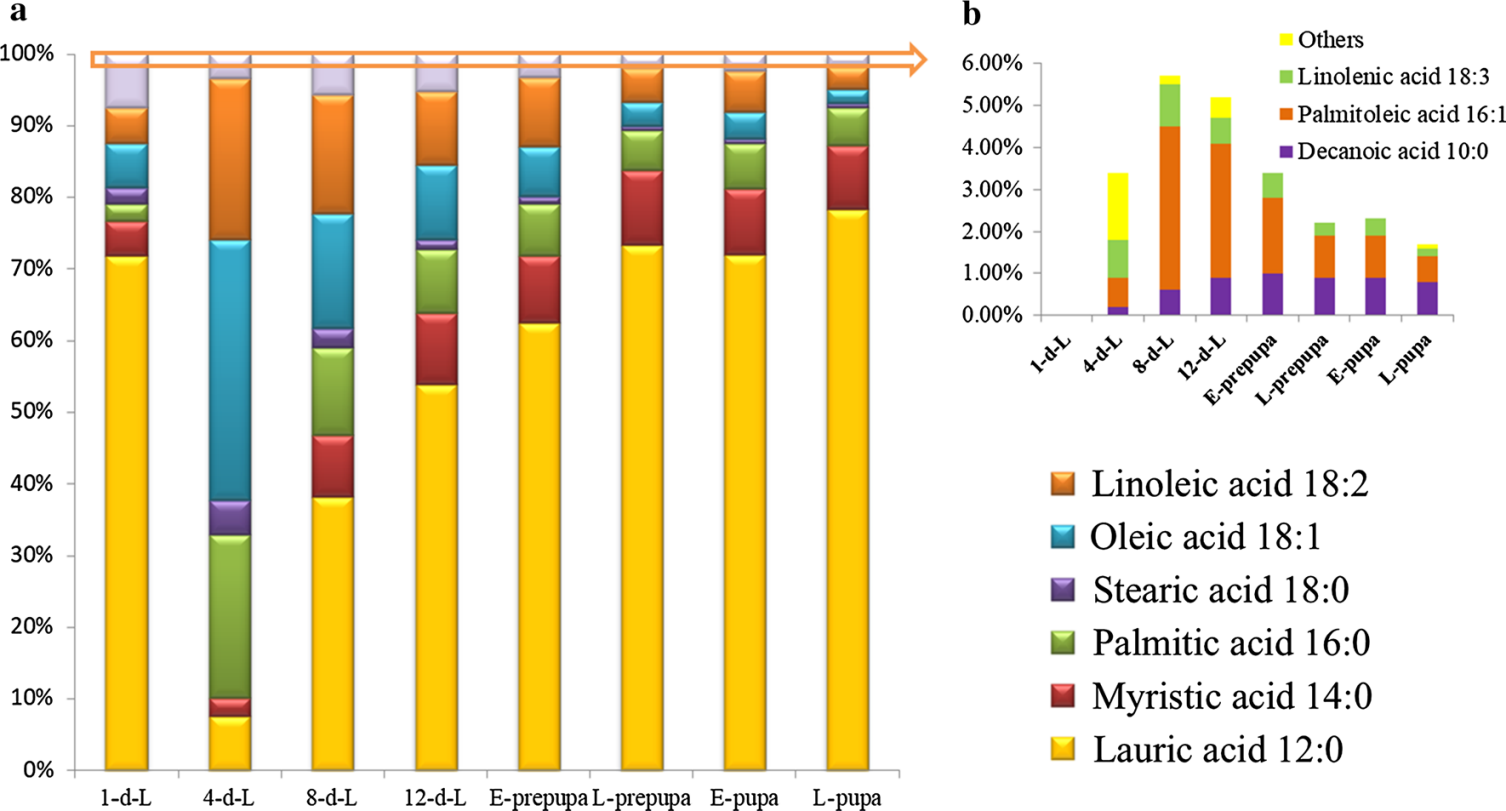
**a** Fatty-acid composition of BSF at different stages of development, **b** fatty acids in small quantity in BSF

Moreover, to explore FA composition in developing BSFL, the dynamic spectra of FAs accumulation in various development phases were analyzed (Fig. 2). The relative proportion of lauric acid (C12:0) was found to be large during developmental stages. The decline of lauric acid (C12:0) was recorded from the 1-d-L stage (72.3%) to the 4-d-L stage (7.7%), at the same time, the proportion of palmitic acid (C16:0), oleic acid (C18:1) and linoleic acid (C18:2) was increased. Whereas the lauric acid (C12:0) showed a rapid increase from 4-d-L to L-prepupa, with a peak value at L-pupa (75.5%), while the proportion of oleic acid (C18:1), palmitic acid (C16:0) and linoleic acid (C18:2) were decreased from 4-d-L to L-prepupa. Moreover, the fluctuation of myristic acid (C14:0) and oleic acid (C18:0) was recorded from 4-d-L to L-pupa. It was noted 90.4% FAs present in developing BSF being short-chain FAs such as C14:0 (myristic acid), C16:0 (palmitic acid) and C12:0 (lauric acid); therefore, it was concluded that BSFL achieves rapid fat accumulation by synthesizing short-chain FAs early in its development, which makes them an ideal feedstock for high-performance biodiesel production.

### Illumina sequencing and de novo assembly of developmental BSF

To investigate the molecular regulatory mechanism of rapid fat accumulation in developing BSF was investigated; therefore, RNA was extracted from E-egg, L-egg, 1-d-L, 4-d-L, 8-d-L, 12-d-L, E-prepupa, L-prepupa, E-pupa, L-pupa, F-adult, and M-adult. The 24 cDNA libraries were constructed and deeply sequenced by the Illumina HiSeq™X Ten system with two replicates in each stage, generating 218,295,450,000 nt from these RNA-Seq samples. After the filtering step, an average of 60,637,625 nt was obtained as clean reads in each sample, with an average Q20 percentage of 97.44% and an average GC percentage of 38.4% (Additional file 1: Table S1). After assembly, 70,475 unigenes were obtained with an N50 of 1749 nt, with the total length of unigenes being 74,988,057 nt and the average length of unigenes being 1064 nt (Additional file 2: Table S2). The length of unigene sequences ranged mainly from 300 to 3000 nt, with 28,121 (39.9%) in the range of 1000 to 3000 nt, 11,335 (16.08%) longer than 2000 nt, and the number of unigenes decreased as the length of sequences increased (Additional file 3: Figure S1). These results indicated that the assembly is of high quality. All clean reads were deposited in the National Center for Biotechnology Information (NCBI) Short Read Archive (SRA) database under accession number PRJNA506627.

### Functional annotation and classification of BSF unigenes

To investigate the function of assembled unigenes in developing BSF, a total of 70,475 unigenes were matched to public databases, including NR (NCBI non-redundant protein sequences), Gene Ontology (GO), Swiss-Prot (a manually annotated and reviewed protein sequence database), Kyoto Encyclopedia of Genes and Genomes (KEGG), Clusters of Orthologous Groups of proteins (COG), and NT (NCBI non-redundant nucleotide sequences). A total of 41,375 (58.7%) unigenes had matches with known genes (Table 1), with 37,960 (53.8%), 24,500 (34.7%), 29,277 (41.5%), 25,758 (36.5%), 19,406 (27.5%), and 19,108 (27.1%) matches in NR, GO, Swiss-Prot, KEGG, COG and NT, respectively. There were 29,100 (41.3%) unigenes with no matches, which may be due to tissue-specific novel genes or short sequences that do not contain a characterized protein domain to have BLAST hits.

**Table 1:** Functional annotation of BSF unigenes in public databases

Database	Number of annotated unigenes (*n*)	Percentage of annotated unigenes (%)
NR	17,960	53.86
Swiss-Prot	29,277	41.54
KEGG	25,758	36.55
GO	24,500	34.76
COG	19,406	27.53
NT	19,108	27.11
Total	41,375	58.71

Similarity analysis between the unigenes of BSF and NR was performed using BLAST (Additional file 4: Figure S2). The results exhibited that 51.9% of the annotated unigenes had strong homology with *e* value below 1*e*^−45^ (Additional file 4: Figure S2. 4A). There were 36.6%, 26.2% and 14.1% of putative proteins showing 40–60%, 60–80% and 80–100% of similarity with known proteins in NR, respectively (Additional file 4: Figure S2. 4B). From the species distribution of NR BLAST matches (Additional file 4: Figure S2. 4C), 24.1% of unigenes had strong homology with *Drosophila*. When compared to other species within Diptera, 12.3% of unigenes had matches to sequences from *Aedes aegypti*, followed by *Culex pipiens quinquefasciatus* (6.7%) and *Anopheles gambiae* PEST (5.4%) (Additional file 4: Figure S2. 4C), which are all mosquitoes.

A total of 24,500 unigenes were categorized into three main GO functional categories (biological process, cellular component, and molecular function) and 61 sub-categories (Additional file 5: Figure S3). Among the 61 sub-categories, ‘cellular process’ and ‘single-organism process’ were the two largest sub-categories that contained 17,197 (70.2%) unigenes and 15,010 (61.3%) unigenes, respectively. Large amounts of unigenes belonged to other sub-categories such as ‘cell’, ‘cell part’ and ‘metabolic process’, which contained 14,663 (59.8%) unigenes, 14,621 (59.7%) unigenes and 13,921 (56.8%) unigenes, respectively. These results revealed that many metabolic activities occur during the development of BSF. Only a few unigenes belonged to the sub-categories of ‘chemoattractant activity’, ‘chemorepellent activity’ and ‘nutrient reservoir activity’.

A total of 19,406 (27.5%) unigenes were categorized into 25 COG classifications (Additional file 6: Figure S4). Among these classifications, the cluster ‘General function prediction only’ represented the largest group, which contained 6218 (32.1%) unigenes. This indicated the existence of a large number of unknown genes in BSF, which may have a excellent exploration potential. The second largest group was ‘Carbohydrate transport and metabolism’ with 3215 (16.6%) unigenes, followed by ‘Transcription’ with 2850 (14.7%) unigenes, and ‘Posttranslational modification, protein turnover, chaperones’ with 2476 (12.7%) unigenes. Only six unigenes were assigned to ‘Nuclear structure’ (0.03%), which was the smallest group.

A total of 25,758 unigenes were categorized into five KEGG categories (A: Cellular Processes, B: Environmental Information Processing, C: Genetic Information Processing, D: Metabolism, and E: Organismal Systems), 41 sub-categories and 259 pathways (Additional file 7: Figure S5). Among the five categories, ‘Metabolism’ had a significantly larger number of unigenes than other categories, which contained 9134 (35.5%) unigenes, followed by ‘Organismal Systems’ with 7934 (30.8%) unigenes, ‘Cellular Processes’ with 5480 (21.3%) unigenes, ‘Genetic Information Processing’ with 4640 (18.1%) unigenes, and ‘Environmental Information Processing’ with 3829 (14.8%) unigenes. Among the 41 sub-categories, ‘Digestive system’ contained the largest number of 2124 (8.3%) unigenes, which may be explained by the fact that BSF can efficiently utilize the wastes, followed by ‘Signal transduction’ with 2051 (7.9%) unigenes, and ‘Transport and catabolism’ with 2032 (7.9%) unigenes. The smallest group was ‘Biosynthesis of other secondary metabolites’, which contained 53 (0.21%) unigenes.

**Fig. 3:**
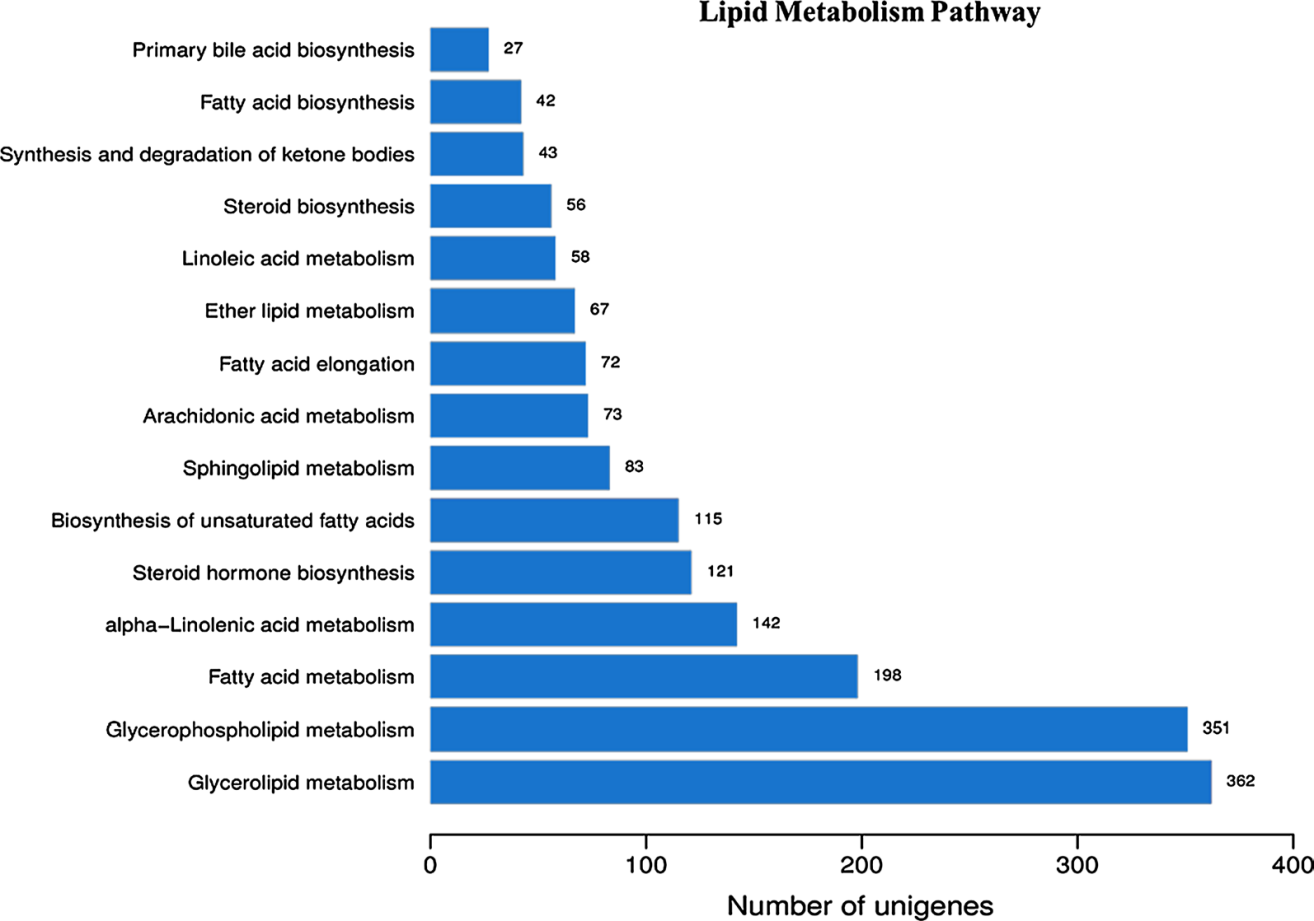
Statistics of BSF unigene distribution in 15 lipid metabolic canonical pathways

To investigate the fat accumulation mechanism of BSF, we screened the results of KEGG pathway annotations. A total of 1810 (7.1%) unigenes were matched into 15 canonical pathways of lipid metabolic from among 259 pathways (Fig. 3). Among these 15 lipid metabolic canonical pathways, ‘Glycerolipid metabolism’ had the largest number of 362 unigenes, followed by ‘Glycerophospholipid metabolism’ with 351 unigenes, ‘Fatty-acid metabolism’ with 198 unigenes, ‘alpha-Linolenic acid metabolism’ with 142 unigenes, ‘Steroid hormone biosynthesis’ with 121 unigenes, and ‘Biosynthesis of unsaturated fatty acids’ with 115 unigenes. The pathway ‘Primary bile acid biosynthesis’ contained only 27 unigenes, while other pathways such as ‘FA biosynthesis’ contained 42 unigenes, and ‘FA elongation’ contained 72 unigenes.

### Analysis of differentially expressed unigenes of developing BSF

**Fig. 4:**
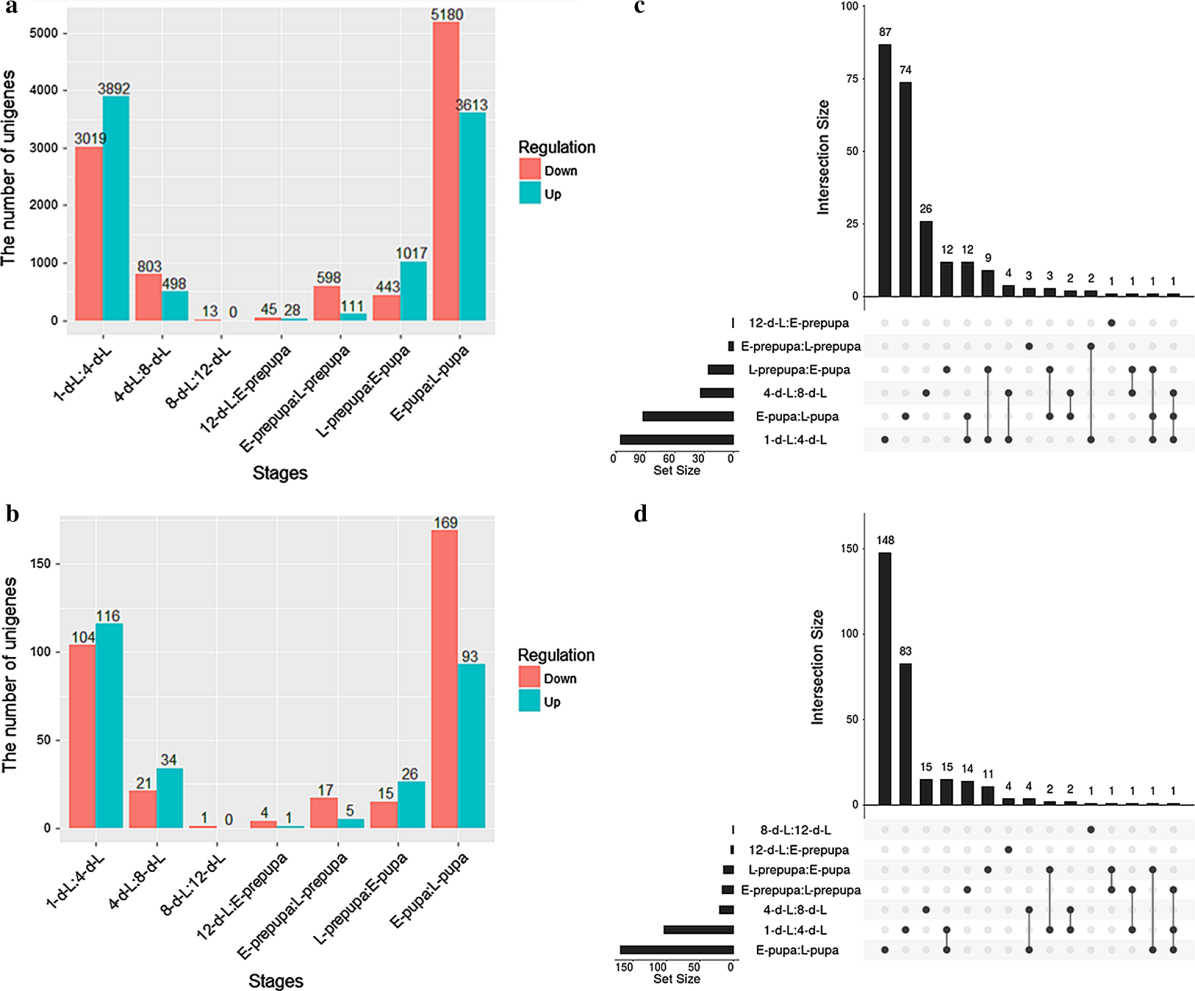
Number and distribution of differentially expressed unigenes that are involved in. **a** Developing BSF. **b** Lipid metabolism in developing BSF. **c** Distribution of up-regulated unigenes that are associated with lipid metabolism in developing BSF. Rows represent the sets of differentially expressed unigenes in different developmental stages. Columns represent their intersections. A vertical black line connects black circles to emphasize column-based relationships. A single black circle represents a set that is not part of the intersection. The size of the intersection is shown in a vertical bar chart placed above each column. The horizontal bar chart represents the size of each set. **d** Distribution of down-regulated unigenes that are associated with lipid metabolism in developing BSF

The differential expression patterns of specific unigenes that are associated with BSF fat accumulation were investigated by calculating the ‘fragment per kilobase per million’ (FPKM) value. A false discovery rate (FDR) ≤ 0.05 was imposed with the absolute value of log_2_ (ratio) ≥ 1 to screen differentially expressed unigenes from all assembled BSF unigenes (Additional file 8: Table S3). The differentially expressed unigenes were matched into the GO database (Additional file 9: Table S4) and the KEGG database (Additional file 10: Table S5). Differentially expressed unigenes were concentrated during the early stages and the late stages, with 6911 of them identified during early stages (1-d-L and 4-d-L) and 8793 of them identified during late stages (E-pupa and L-pupa), while very few differentially expressed unigenes were identified at 8-d-L and 12-d-L (Fig. 4a). When the differentially expressed unigenes that are involved in lipid metabolism were screened and analyzed, they were also concentrated during the early stages and the late stages, with 220 of them identified at 1-d-L and 4-d-L, and 262 of them identified at E-pupa and L-pupa (Fig. 4b). As a result, lipid metabolism occurs mainly during the early stages and the late stages.

Five unigenes were found to have sustained up-regulation in early stage (1-d-L, 4-d-L and 8-d-L), while four unigenes were found to have sustained up-regulation in late stage (L-prepupa, E-pupa and L-pupa) (Fig. 4c). The five unigenes that have sustained up-regulation in early stage were related to triacylglycerol lipase (lip, EC:3.1.1.3) (Unigene22405_All), lipoprotein lipase (LPL, EC:3.1.1.34) (Unigene25391_All), carboxylesterase 1 (CES1, EC:3.1.1.1) (CL7976.Contig2_All), glucuronosyltransferase (UGT, EC:2.4.1.17) (CL182.Contig2_All), and beta-galactosidase (GLB1, EC:3.2.1.23) (CL2559.Contig1_All). The lip and LPL are involved mainly in the triacylglycerol degradation pathway. GLB1 can catalyze the decomposition of lactose to d-galactose and alpha-d-glucose, which can be further decomposed to provide energy. UGT can catalyze the conversion of UDP-glucuronate and beta-d-glucuronoside.

Among the four unigenes that have sustained up-regulation in late stage, two were related to aldehyde reductase (AKR1B, EC:1.1.1.21) (CL7623.Contig2_All, CL3836.Contig1_All), one was related to elongation of very long-chain fatty-acid protein 4 (ELOVL4, EC:2.3.1.199) (CL8829.Contig3_All), and one was related to (3R)-3-hydroxyacyl-CoA dehydrogenase/3a,7a,12a-trihydroxy-5b-cholest-24-enoyl-CoA hydratase/enoyl-CoA hydratase 2 (HSD17B4, EC:1.1.1.-4.2.1.107 4.2.1.119) (CL2509.Contig4_All). ELOVL4 and HSD17B4 are associated with biosynthesis of unsaturated fatty acids and fatty-acid elongation.

The distribution of down-regulated unigenes that are involved in lipid metabolism was also analyzed (Fig. 4d). Two unigenes were found to have sustained down-regulation in early stage (1-d-L, 4-d-L and 8-d-L). Among them, one unigene was related to AKR1B, and another unigene was related to diacylglycerol kinase (ATP) (DGK, EC:2.7.1.107) (CL3020.Contig2_All).

Meanwhile, two unigenes were found to have sustained down-regulation in late stage (E-prepupa, L-prepupa, E-pupa and L-pupa). Among them, one was related to 3-hydroxy acid dehydrogenase/malonic semialdehyde reductase (ydfG, EC:1.1.1.381 1.1.1.-) (CL10341.Contig4_All), with sustained down-regulation in E-prepupa, L-prepupa and E-pupa. The enzyme ydfG is a member of the 3-hydroxyacyl-CoA dehydrogenase family and can reduce malonic semialdehyde with NADPH to 3-hydroxypropionate. The other unigene was related to aldehyde dehydrogenase (NAD+) (ALDH, EC:1.2.1.3) (CL258.Contig4_All), with sustained down-regulation in L-prepupa, E-pupa and L-pupa.

### Expression patterns of enzymes involved in pyruvate and acetyl-CoA formation in developing BSF

To investigate the expression patterns of genes associated with pyruvate formation, putative genes that are related to enzymes required for glycolysis were obtained from Illumina sequencing analysis. Among the 122 putative genes that are associated with glycolysis in BSF, 12 of them were related to hexokinase (HK, EC:2.7.1.1, *e* value: 6*e*^−9^ to 0), 5 of them were related to ADP-dependent glucokinase (ADPGK, EC:2.7.1.147, *e* value: 2*e*^−18^ to 5*e*^−140^), 12 of them were related to glucose-6-phosphate isomerase (GPI, EC:5.3.1.9, *e* value: 3*e*^−15^ to 0), 16 of them were related to 6-phosphofructokinase 1 (PFK, EC:2.7.1.11, *e* value: 1*e*^−6^ to 0), 3 of them were related to fructose-1,6-bisphosphatase I (FBP, EC:3.1.3.11, *e* value: 7*e*^−50^ to 9*e*^−158^), 5 of them were related to fructose-bisphosphate aldolase, class I (ALDO, EC:4.1.2.13, *e* value: 2*e*^−11^ to 0), 11 of them were related to glyceraldehyde 3-phosphate dehydrogenase (GAPDH, EC:1.2.1.12, *e* value: 1*e*^−14^ to 0), 4 of them were related to phosphoglycerate kinase (PGK, EC:2.7.2.3, *e* value: 2*e*^−19^ to 0), 11 of them were related to 2,3-bisphosphoglycerate-dependent phosphoglycerate mutase (gpmA, EC:5.4.2.11, *e* value: 3*e*^−8^ to 2*e*^−147^), 2 of them were related to 2,3-bisphosphoglycerate-independent phosphoglycerate mutase (gpmI, EC:5.4.2.12, *e* value: 3*e*^−29^ to 6*e*^−56^), 6 of them were related to enolase (ENO, EC:4.2.1.11, *e* value: 1*e*^−12^ to 0), and 22 of them were related to pyruvate kinase (PK, EC:2.7.1.40, *e* value: 1*e*^−7^ to 0).

**Fig. 5:**
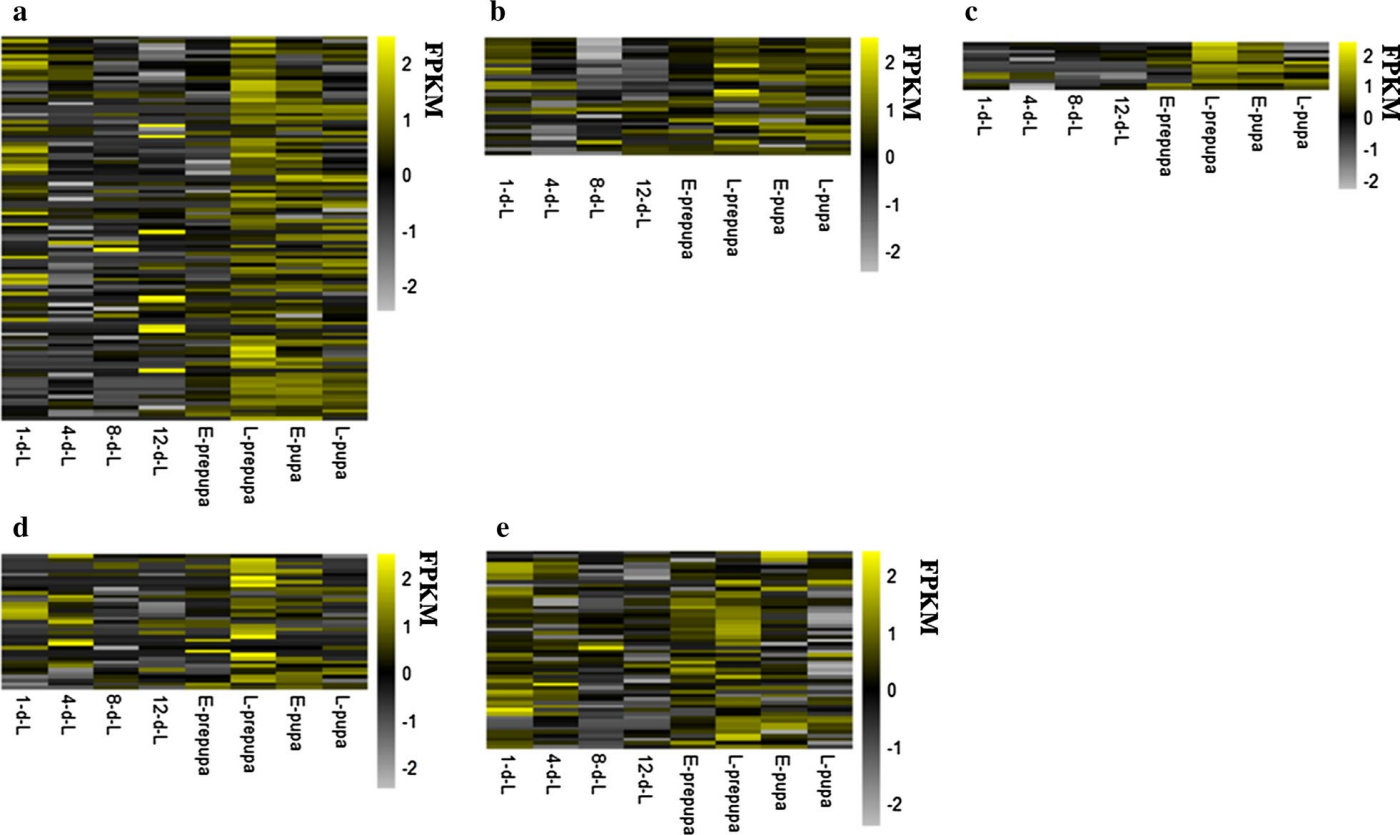
Temporal expression profile of putative genes that are involved in fat accumulation in developing BSF larvae by z-score normalized FPKM. **a** Temporal expression profile of putative genes related to glycolysis. **b** Temporal expression profile of putative genes related to PDC subunits E1-α, E1-β, E2, and E3. **c** Temporal expression profile of putative genes in citrate–pyruvate cycle. **d** Temporal expression profile of putative genes for fatty-acid biosynthesis. **e** Temporal expression profile of putative genes for TAG biosynthesis. Expression values in heatmap are shown as z-score normalized FPKM

**Fig. 6:**
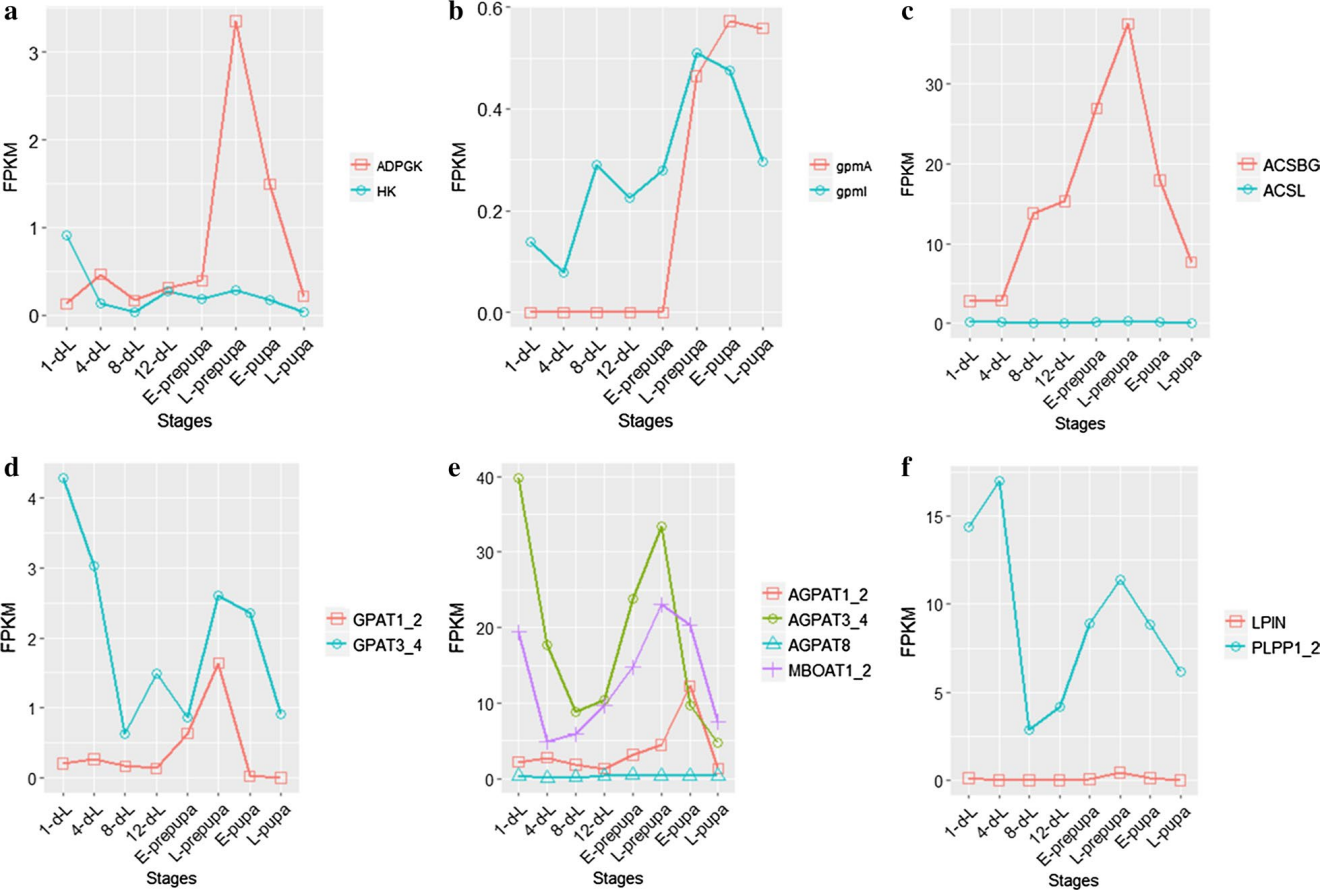
Temporal profile of FPKM of putative genes that encode enzymes involved in crude fat accumulation. **a** The temporal profile of HK and ADPGK; **b** The temporal profile of gpmA and gpmI; **c** The temporal profile of ACSBG and ACSL; **d** The temporal profile of GPAT1_2 and GPAT3_4; **e** The temporal profile of AGPAT1_2, AGPAT3_4, AGPAT8 and MBOAT1_2; **f** The temporal profile of LPIN and PLPP1_2

Temporal transcript analysis was performed to examine the dynamic expression patterns of putative genes that are involved in glycolysis. The putative genes from glycolysis pathway were highly expressed during early and late stages of BSFL development (Fig. 5a). When compared the temporal profile of FPKM for putative genes encoding isozymes HK, ADPGK, gpmA and gpmI that are involved in glycolysis, HK had higher expression in early stage, while ADPGK was up-regulated at L-prepupa (Fig. 6a), meanwhile, the expression patterns of gpmI showed more consistent with CF accumulation patterns (Fig. 6b).

Pyruvate dehydrogenase complex (PDC) is an important enzyme for acetyl-CoA formation. There were 8, 7, 6 and 11 putative genes that are related to PDC subunits E1-α, E1-β, E2 and E3, respectively, with *e* value ranging from 6*e*^−16^ to 3*e*^−170^, 5*e*^−25^ to 4*e*^−172^, 5*e*^−6^ to 0 and 2*e*^−6^ to 0, respectively. The expression levels of these genes gradually decreased before 8-d-L, but increased from 8-d-L, with the highest expression level at L-prepupa (Fig. 5b). Since the putative genes that are associated with acetyl-CoA formation are also putative genes involved in glycolysis (Fig. 5a), the PDC subunits can respond to BSFL development, with acetyl-CoA production for CF accumulation occurring mainly in early stage. Although the PDC subunits had high expression level in late stages such as E-pupa and L-pupa, the CF content did not increase significantly. As a result, acetyl-CoA was used to provide energy rather than CF accumulation in late stage.

### Expression patterns of enzymes involved in acetyl-CoA transportation and FA biosynthesis in developing BSF

By Illumina sequencing analysis, 8 and 5 putative genes were identified relation to the citrate synthase (CS, EC:2.3.3.1, *e* value: 3*e*^−6^ to 0) and ATP-citrate lyase (ACLY, EC:2.3.3.8, *e* value: 0). Acetyl-CoA can be catalyzed by CS to produce citric acid by condensation of oxaloacetic acid, and citrate is preferentially exported to the cytosol via the tricarboxylate transporter. Similarly, we analyzed the dynamic expression patterns of putative genes that are associated with acetyl-CoA transportation, the putative genes of CS and ACLY had similar expression patterns as pyruvate and acetyl-CoA biosynthesis (Fig. 5c).

The putative genes involved in FA biosynthesis in developing BSF were identified by Illumina sequencing analysis. There are 11 putative genes were related to encoding acetyl-CoA carboxylase (ACC, EC: 6.4.1.2, *e* value: 2*e*^−7^ to 0), 22 that were related to fatty-acid synthase, animal type (FASN, EC:2.3.1.85, *e* value: 2*e*^−6^ to 0), 4 that were related to [acyl-carrier-protein (ACP)] *S*-malonyltransferase (FabD, EC:2.3.1.39, *e* value: 9*e*^−14^ to 2*e*^−156^), and 4 that were related to 3-oxoacyl-[ACP] synthase II (FabF, EC:2.3.1.179, *e* value: 3*e*^−6^ to 7*e*^−153^). Since the first step of FA biosynthesis were catalyzed by ACC, ACC has been considered as a major rate-controlling enzyme in this pathway. Additionally, lauric acid (C12:0) is the main component of BSFL FAs, this phenomenon indicated that FASN possesses the function to produce lauric acid (C12:0). Interestingly, when the unigenes were matched into the canonical pathways of fatty-acid biosynthesis, we observed that FASN catalyzes a series of reactions in this pathway (Additional file 11: Figure S6). As shown by temporal transcript analysis, the putative genes involved in FA biosynthesis had higher expression in early stage and in late stage (Fig. 5d).

### Expression patterns of enzymes involved in triacylglycerol synthesis in developing BSF

Triacylglycerol (TAG) biosynthesis begins with acyl-CoA formation. Two isozymes were identified by Illumina sequencing analysis in the acyl-CoA biosynthesis pathway, with 10 putative genes that were related to long-chain-fatty-acid-CoA ligase (ACSBG, EC:6.2.1.3, *e* value: 7*e*^−11^ to 0) and 6 putative genes that were related to long-chain acyl-CoA synthetase (ACSL, EC:6.2.1.3, *e* value: 1*e*^−17^ to 0). ACSBG had high expression level in developing BSFL from the temporal transcript analysis of ACSBG and ACSL (Fig. 6c).

By Illumina sequencing analysis, 54 putative genes for TAG biosynthesis were identified. Temporal transcript analysis showed that the putative genes for TAG biosynthesis were highly expressed during early stages (1-d-L to 4-d-L) and late stages (E-prepupa to E-pupa) (Fig. 5e). Since differential expression analysis indicated that TAG degradation occurs mainly during the early stages, these results indicated that rapid TAG accumulation occurs mainly during the late stages.

Glycerol-3-phosphate *O*-acyltransferase (GPAT) catalyzes the first step of TAG biosynthesis. It plays a critical role in the conversion of glycerol 3-phosphate and acyl-CoA to 1-acyl-sn-glycerol 3-phosphate. From among the putative genes that are related to GPAT, 13 of them were related to glycerol-3-phosphate *O*-acyltransferase 1/2 (GPAT1_2, EC:2.3.1.15, *e* value: 2*e*^−10^ to 6*e*^−133^), and 4 of them were related to glycerol-3-phosphate *O*-acyltransferase 3/4 (GPAT3_4, EC:2.3.1.15, *e* value: 2*e*^−124^ to 6*e*^−177^). GPAT3_4 had high expression level from the temporal transcript analysis of GPAT1_2 and GPAT3_4 (Fig. 6d).

In the second step of TAG biosynthesis, an additional FA is transferred to 1-acyl-sn-glycerol 3-phosphate by the family members of 1-acylglycerol-3-phosphate acyltransferase (AGPAT) to produce 1,2-diacyl-sn-glycerol 3-phosphate. Interestingly, three isozymes of AGPAT and one putative acyltransferase were identified to catalyze this step, with 11 putative genes that were related to AGPAT1_2 (EC:2.3.1.51, *e* value: 2*e*^−14^ to 5*e*^−108^), 3 putative genes that were related to AGPAT3_4 (EC:2.3.1.51 2.3.1.-, *e* value: 3*e*^−8^ to 1*e*^−123^), 1 putative gene that was related to AGPAT8 (EC:2.3.1.51 2.3.1.-, *e* value: 3*e*^−8^), and 1 putative gene that was related to lysophospholipid acyltransferase 1/2 (MBOAT1_2, *e* value: 6*e*^−152^, EC:2.3.1.51 2.3.1.-). Temporal transcript analysis for the isozymes of AGPAT and MBOAT1_2 showed that the expression patterns of AGPAT3_4 are consistent with the ones from putative genes involved in TAG biosynthesis (Fig. 6e).

In the third step of TAG biosynthesis, phosphatidate is dephosphorylated to provide 1,2-diacylglycerol (DAG) for the biosynthesis of TAG. Two isozymes of phosphatidate phosphatase were identified by Illumina sequencing analysis, with 11 putative genes that were related to phosphatidate phosphatase (PLPP1_2_3, EC:3.1.3.4, *e* value: 2*e*^−27^ to 8*e*^−105^), and 8 putative genes that were related to phosphatidate phosphatase LPIN (LPIN, EC:3.1.3.4, *e* value: 3*e*^−12^ to 0). Temporal transcript analysis of PLPP1_2_3 and LPIN showed that PLPP1_2_3 had high expression level, and the expression patterns are consistent with the ones from putative genes involved in TAG biosynthesis (Fig. 6f).

In the last step of TAG biosynthesis, acyl-CoA: diacylglycerol acyltransferase (DGAT) is used to synthesize FA into triglycerides. In this study, only two putative genes were identified to be related to diacylglycerol *O*-acyltransferase 1 (DGAT1, EC:2.3.1.20 2.3.1.75 2.3.1.76, *e* value: 0) in developing BSF; the result indicated DGAT1 is specific to TAG biosynthesis in BSF.

### Experimental validation and analysis of key genes involved in BSF fat accumulation

**Fig. 7:**
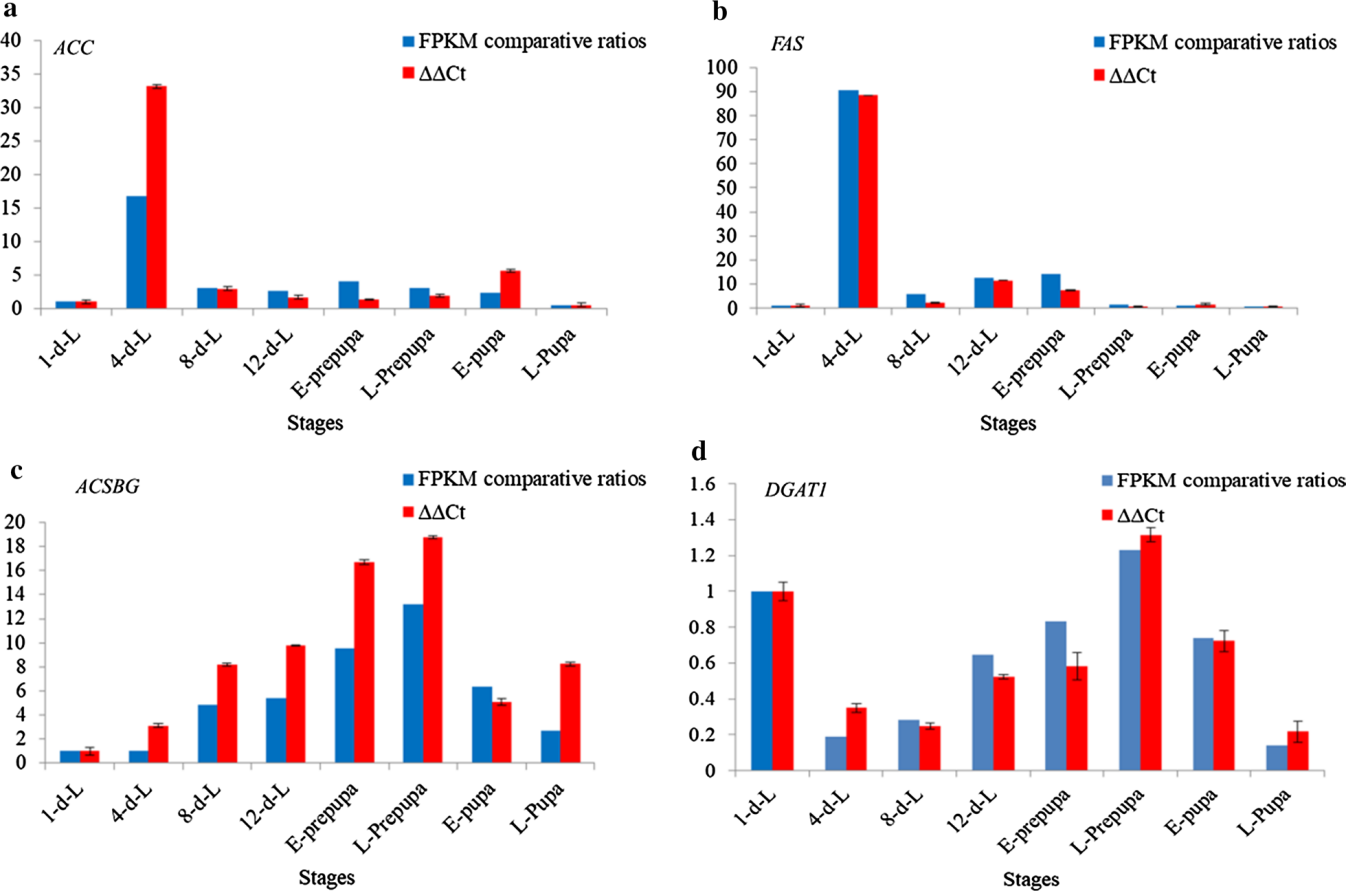
Quantitative RT-PCR validation of four candidate lipid-related unigenes (**a** ACC, **b** FAS, **c** ACSBG and **d** DGAT1) of BSF. The comparative FPKM ratio and 2^−ΔΔCt^ at 1-d-L are used as a control for normalization

To assess the accuracy of sequencing and assembly of the BSF transcriptome, the relative expression levels and temporal transcript patterns of the putative genes which involved in fat accumulation were analyzed. Four putative genes of vital enzymes, including *FAS*, *ACC*, *ACSBG* and *DGAT1*, were selected for qRT-PCR (Additional file 12: Table S6). The results from qRT-PCR showed that the relative expression levels of these selected genes were mostly consistent with the FPKM comparative ratios (with 1-d-L as the control) (Fig. 7). These results indicated that the unigene assembly is accurate and reliable, and it is feasible to use the DESeq method to select differentially expressed gene. Both enzymes *ACC* and *FAS* that are involved in FA biosynthesis had high expression level at the 4-d-L stage.

## Discussion

BSFL develops on organic wastes, reducing environmental pollution and converting waste into biodiesel from the larval fat body. Therefore, BSF fat as feedstock for biodiesel production is studied widely; moreover, the investigation of the molecular regulatory basis of rapid fat accumulation by BSF is pivotal for biodiesel development. Furthermore, the identification of vital genes and regulators that are involved in fat accumulation will optimise the conversion to biodiesel. The fat content and FA composition of BSFL in different stages were compared (Figs. 1, 2). Larvae early in the development process have not accumulated fat, while those late in the development process (i.e., E-pupa and L-pupa) have utilized fat reserves to complete metamorphosis, which is in agreement with previous results [11]; meanwhile, the sudden decline of lauric acid from the 1-d-L stage (72.3%) to the 4-d-L stage (7.7%) may be due to its utilization as stored energy at 1-d-L. The dynamic variation of lauric acid (C12:0) during BSFL development indicated that it could be important in energy and metabolism. Similar results showed that other flies (Diptera) were characterized to have a very high proportion of lauric acid (C12:0), palmitoleic acid (C16:1), and decanoic acid (C10:0) [23, 24].

The fat accumulation and regulation in BSF at the molecular level, sequencing of BSF transcriptome were performed to comprehensively analysis the transcriptome profile in developing BSF. Since the reference genome of BSF is not available, to obtain the reliable results of assembly and annotation, exception the eight stages (1-d-L, 4-d-L, 8-d-L, 12-d-L, E-prepupa, L-prepupa, E-pupa and L-pupa) that we investigated the dynamic patterns of fat content and FA composition, four additional stages (E-egg, L-egg, F-adult and M-adult) are also added, resulting in a total of twelve stages was performed deep sequencing, the greater sequencing depth and accurate sequence reads provide reliable and effective data of BSF transcriptome profile.

The analysis of differentially expressed gene is a powerful approach to identify the key regulator underlying important biological processes. In this study, we identified five enzymes (lip, LPL, CES1, UGT and GLB1) that have sustained up-regulation in early stage, three enzymes (AKR1B, ELOVL4 and HSD17B4) that have sustained up-regulation in late phase (Fig. 4c). It is noteworthy that the enzymes up-regulated expression in early stage primary involvement lipid and carbohydrate metabolism, these results indicated that lip, LPL, CES1, UGT and GLB1 play vital role in energy supply. The previous researches showed that UGT is a very important detoxifying enzyme that participates in host defense against endogenous toxins and xenobiotic chemicals, and is discovered in various living organisms from bacteria to humans [25]. In humans, CES1 was found to have fatty-acid ethyl ester (FAEE) synthesizing activity, in which long-chain fatty acids are transesterified with ethanol to generate FAEEs [26]; moreover, it was formerly noted that CES1 appears to be participated in some processes important to cell biology, like cholesterol trafficking [27]. In addition to its catalytic actions, it was recorded that CES1 appears to play critical role for protein release and retention from the endoplasmic reticulum (ER) [28], and the UGT phase II drug metabolism enzymes can form a complex with CES1 to hold UGTs in the ER lumen [29]. These results indicated that UGT and CES1 play vital roles in BSFL lipid metabolism and defense against toxins. AKR1B was proved to play a significant role in the biosynthesis of ascorbic acid and develop scurvy [30]. Whereas in this investigation AKR1B was discovered sustained down-regulation in early stage and supported up-regulation in late stage. Since it is well known that ascorbic acid is an antioxidant that suppresses osteoclast activity, the ALR1B may play a vital role in BSF eclosion. Although ELOVL4 and HSD17B4 were proved involvement in fatty-acid elongation and biosynthesis of unsaturated fatty acids, and both enzymes exhibited up-regulation in late stage. Whereas increase in the content of long-chain FA and unsaturated fatty acids (Fig. 2) was not observed, because there is not enough substrate for the biosynthesis of long-chain fatty acids and unsaturated fatty acids, as BSF stops eating from the prepupa stage [31].

The down-regulated unigenes that are associated with lipid metabolism was also analyzed (Fig. 4d). Two enzymes (ydfG and ALDH) were found to have sustained down-regulation in late stage (E-prepupa, L-prepupa, E-pupa and L-pupa). Although the major function of ALDH is NAD(P)+-dependent aldehyde oxidation, previous studies indicated that ALDHs exhibit multiple functions, such as ALDH1A1, ALDH2, ALDH3A1 and ALDH4A1 are known to catalyze ester hydrolysis, suggesting that ALDHs may have more than one catalytic role [32]. Since both ydfG and ALDH convert aldehydes into acids in developing BSFL, the down-regulation of ydfG and ALDH in late-stage indicated that the conversion of aldehydes to acids was important for lipid metabolism in early and middle stage.

With pyruvate and acetyl-CoA as the substrate for FA biosynthesis, the putative genes from pyruvate and acetyl-CoA formation pathway and acetyl-CoA transportation pathway showed similar expression patterns (Fig. 5a–c). The substrate formation and transportation for FA biosynthesis in the early stage may play an essential role in fat accumulation in developing BSFL. The putative genes encoding isozymes from pyruvate formation pathway were identified, the first step in metabolism of glucose is phosphorylation that is catalyzed by HK and ADPGK [33, 34], the temporal profile of HK and ADPGK suggested that HK may play a vital role in pyruvate formation and fat accumulation, while ADPGK plays an essential role in energy supply, because BSF needs energy for tissue differentiation and eclosion in late stages (Fig. 6a), whereas the expression patterns of gpmI are consistent with CF accumulation, and gpmI may play an important role in glycolysis (Fig. 6b).

Several putative genes from FA biosynthesis in developing BSF were also identified. The analysis of canonical pathways of fatty-acid biosynthesis showed that FASN catalyzes a series of reactions in this pathway (Additional file 11: Figure S6), this phenomenon indicated that FASN plays a significant role for FA biosynthesis in developing BSF. Although the putative genes from FA biosynthesis pathway had higher expression level in late stage, CF content did not increase. Since BSF does not eat at the prepupa stage, there is not enough substrate to accumulate fat. At the pupa stage, energy is needed for tissue differentiation, and fat is consumed to provide energy. Although the putative genes had high expression level in E-pupa to L-pupa stages, CF content is low.

In TAG biosynthesis pathway, several iszymes including ACSBG, ACSL, GPAT1_2, GPAT3_4, AGPAT1_2, AGPAT3_4, AGPAT8, MBOAT1_2, PLPP1_2_3 and LPIN were identified, the comparison of temporal profile showed that ACSBG, GPAT3_4, AGPAT3_4 and PLPP1_2_3 had similar and high expression levels (Fig. 6), these enzymes may play an essential role in TAG biosynthesis pathway.

The putative genes from pyruvate and acetyl-CoA formation pathway, acetyl-CoA transportation pathway, FA and TAG biosynthesis pathway showed similar expression patterns (Fig. 5), as above-mentioned the temporal profile of vital genes involved in fat accumulation, these results indicated BSF possesses the regular fat metabolism pattern, and the fat accumulation in early stages play an important role in BSF development.

**Fig. 8:**
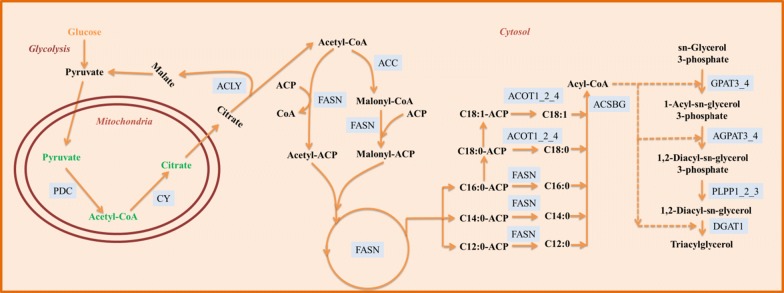
Characterization of the fat accumulation model in developing BSF for the regulation of enzymes in oil biosynthesis. The identified routes of fat accumulation are based on transcriptome data from Illumina sequencing and temporal expression analysis

The expression profiles of metabolic enzymes that are involved in the biosynthesis of acetyl-CoA, FA and TAG were analyzed to investigate the fat accumulation mechanism in BSF. A detailed fat accumulation model was constructed from our results (Fig. 8). It starts with glycolysis and results in the formation of pyruvate and acetyl-CoA, followed by the transportation of acetyl-CoA to the cytosol. The finding of this investigation will help to understand the molecular regulatory basis of rapid fat accumulation in BSF, and provide a foundation to increase content of fat without compromising growth by metabolic engineering, it is an essential aspect of advancing the economic feasibility of BSF waste conversion technology. It was previously noted the knockdown of a multifunctional lipase/phospholipase/acyltransferase in the diatom *Thalassiosira pseudonana* increased lipid content without affecting growth [35]. Moreover, to redirect carbon flux toward fat biosynthesis, the deletion of glycerol-3-phosphate dehydrogenase isomer in *Yarrowia lipolytica* exhibited a threefold remarkably increase in lipid content [36]. Thus, the gene data sets were developed in this investigation lay a foundation for metabolic engineering to increase the content of fat in BSF and also provide a foundation to develop molecular markers for fat breeding studies.

## Conclusion

The dynamic patterns of fat accumulation and FA compositions in different development stages of BSFL were analyzed. It was noted that the E-prepupa showed the highest fat content during BSF metamorphosis. Moreover, FA analysis indicated that lauric acid (C12:0) is the major component in BSF fat body. Furthermore, 24 samples selected for in-depth transcriptome sequencing analysis, a total of 70,475 unigenes were assembled with an average length of 1064 nt from clean reads. By applying the DESeq method, 14,597 differentially expressed unigenes were identified.

The expression profiles of metabolic enzymes that are involved in the biosynthesis of acetyl-CoA, FA and TAG were analyzed to investigate the fat accumulation mechanism in BSF. The regulatory enzymes that play important roles in fat accumulation in BSF, including HK, ADPGK, PFK, CS, ACLY, ACC, FASN, ACSBG, AGPAT3_4, PLPP1_2 and DGAT1 were characterized (Additional file 13: Table S7). The FA and TAG biosynthesis pathways were reconstructed in BSFL, and the temporal expression levels of four vital genes were validated through quantitative real-time PCR (qRT-PCR).

A detailed fat accumulation model was constructed from our results (Fig. 8). It starts with glycolysis and results in the formation of pyruvate and acetyl-CoA, followed by the transportation of acetyl-CoA to the cytosol. The rapid accumulation of predominantly short-chain FAs are produced from acetyl-CoA and ligated to form long-chain acyl-CoA, lipogenesis process ends with TAG formation. Whereas many genes that are involved in these processes are both highly expressed during early stages (1-d-L to 4-d-L) and late stages (E-pupa to L-pupa), it was noted that the CF content does not increase significantly during late stages. Therefore, most of these processes up to FA biosynthesis occur during the early stages. Moreover, it was observed that the TAG degradation occurs mainly during the early stages by differentially expressed analysis; this evidence proved that the rapid TAG accumulation occurs mainly during the late stages.

The finding of this investigation will help to understand the molecular regulatory basis of rapid fat accumulation in BSF. It will provide a useful resource for functional genomics studies, and enrich the public database. Meanwhile, the gene data sets were developed in this investigation lay a foundation for metabolic engineering to increase the content of fat in BSF and also provide a foundation to develop molecular markers for fat breeding studies.

## Materials and methods

### Insect materials and extraction of BSF fat

The colony of BSF was maintained in a greenhouse at the National Engineering Research Center of Microbial Pesticides, Huazhong Agricultural University (HZAU), Wuhan, Hubei, China, for about 10 years. The commercial chicken feed used to rear BSF was produced by Charoen Pokphand Group, Wuhan, China. The nutritional composition of this chicken feed was listed in (Additional file 14: Table S8).

Fresh eggs of BSF were collected from the colony. The eggs were immediately frozen in liquid nitrogen for 90 s, marked as E-egg (< 12 h), and stored at − 80 °C until they were used. A batch of eggs was placed in incubators at 26 °C with 65–70% relative humidity (RH) for hatching, with neonate larvae developed on the chicken feed. The eggs marked as L-egg (< 72 h) were collected after 2 days from hatching eggs and frozen in liquid nitrogen for 90 s and stored at − 80 °C. The egg hatchings were monitored, and the larvae that hatched within 3 h were collected and frozen in liquid nitrogen for 90 s, marked as one-day-old (1-d-L), and stored at − 80 °C. The remaining neonate larvae were separated into five groups and reared with 150 g of moist chicken feed having 70% water content at 26 °C with 60% RH. Larvae samples at various developmental stages (4-d-L, 8-d-L and 12-d-L) were collected using the previously described method. The newly emerged prepupae were removed promptly every day. Prepupae were collected from the 2nd day after preparation started and frozen in liquid nitrogen for 90 s, marked as E-prepupae, and stored at − 80 °C. The remaining prepupae were left for pupation at 26 °C with 60% RH. The pupae were observed, immediately removed and collected. The freshly emerged pupae were selected and marked as L-prepupae and E-pupa, frozen in liquid nitrogen for the 90 s and stored at − 80 °C. The remaining pupae were monitored for adult emergence. Once adult emerged, late pupae were collected and marked as L-pupa, frozen in liquid nitrogen for 90 s and stored at − 80 °C. The adult female and male flies that emerged within 12 h were selected and marked as F-adult and M-adult, frozen in liquid nitrogen for the 90 s stored at −  80 °C. The fat content and fatty-acid composition of BSF were analyzed concerning China National Standards GB/T 5009.6-2003 and GB/T 17376-2008. In brief, to measure the fat content, the 2 g sample was put into the 50 ml test tube with 10 ml of hydrochloric acid and 8 ml of water. The sample was digested completely in 70–80 °C water bath, the 10 ml ethanol was added into the digested sample, following the digested sample was transferred to a 100 ml mix cylinder with stopper, the cylinder was added of 5 ml petroleum ether–ether mixture and allowed to stand. The supernatant was taken out from the cylinder, the residue was collected and transferred to the flask on a water bath, the flask was evaporated repeatedly to constant weight, finally, the total fat content was measured (GB/T 5009.6-2003). To analysis the fatty-acid composition, 0.5 g sample was put into 50 ml flask with 6 ml of sodium hydroxide methanol solution, the sample was saponified. To obtain the methyl ester solution, seven ml of boron trifluoride was added in the flask and extracted for 3 min. Finally, the FAs were determined by gas chromatography analysis using 0.2 μl of methyl ester solution (GB/T 17376–2008).

### cDNA library preparation and sequence data analysis and assembly

Twelve developmental stages (E-egg, L-egg, 1-d-L, 4-d-L, 8-d-L, 12-d-L, E-prepua, L-prepua, E-pupa, L-pupa, F-adult, and M-adult) in BSF were selected for transcriptome sequencing and analysis. With two biological replicates per stage, 24 samples were constructed. RNA was extracted with Trizol reagent (Vazyme BioTech Co. Ltd, Nanjing, China) and treated with DNase I. The mRNA was isolated by magnetic beads with Oligo (dT) and mixed with the fragmentation buffer to obtain the short fragments. The cDNA synthesis was performed using the mRNA fragments as templates. The short fragments purified from the cDNA synthesis were used for end reparation, single nucleotide A (adenine) addition and adaptors connection. Following, the fragments with suitable length were selected for the PCR amplification as templates; meanwhile, the quantification and qualification of the sample libraries were measured by Agilent 2100 Bioanalyzer and ABI StepOnePlus Real-Time PCR System. Finally, the libraries were sequenced using Illumina HiSeq™ X Ten.

Since the raw reads produced from sequencing machines contain dull reads with adaptors, unknown or low-quality bases, they were filtered before bioinformatics analysis: (1) remove adaptor sequences; (2) remove reads with more than 5% unknown nucleotides; and (3) remove low-quality reads with more than 20% of nucleotides having quality value < 10. Clean reads were assembled by the Trinity software (version: release-20130026) [37].

### Sequence annotation

The unigenes of BSF were annotation by BLAST (version 2.2.26) search against the NR, Swiss-Prot and KEGG database. The Blast2GO program [38] was applied to obtain GO annotation of BSF unigenes, the WEGO software [39] was applied to perform GO functional classification to obtain the distribution of gene function at the macro level. All assembled unigenes were matched to the COG database and further annotated by blastx with *e* value < 0.00001, and NT (NCBI non-redundant nucleotide sequences) by blastn with *e* value < 0.00001.

### Differential expression analysis of unigenes

FPKM values were calculated to evaluate expression levels of unigenes. A standard FDR ≤ 0.05 and the absolute value of log_2_(ratio) ≥ 1 were imposed to screen differentially expressed unigenes in developing BSF.

### qRT-PCR validation

Total RNA was extracted as described in cDNA library preparation. Reverse transcription was performed using HiScript II Q Select RT SuperMix (Vazyme Biotech Co., Ltd, China) according to the manufacturer’s protocol. The amplification primers were designed using the Primer Premier 5.0 software (Premier Biosoft International, Palo Alto, CA, USA), with β-actin as an internal control. Three technical repetitions were performed for qRT-PCR.

## Additional files

**Additional file 1: Table S1.** Raw data.

**Additional file 2: Table S2.** The result of unigenes assemble.

**Additional file 3: Figure S1.** The length distribution of BSF unigenes.

**Additional file 4: Figure S2.** Similarity analysis between BSF unigenes and NR database. **(A)** *e* value (< 1*e*-5) distribution of top BLAST hits for each BSF unigene. **(B)** Similarity (> 17%) of BSF putative proteins with known proteins in NR database. **(C)** Top-hit species distribution of BLAST matches for BSF unigenes.

**Additional file 5: Figure S3.** Gene ontology (GO) classification of BSF unigenes. The left side and the right side of the panel show the percentage of genes and the number of genes that are classified to the three main categories, including biological process, cellular component, and molecular function.

**Additional file 6: Figure S4.** COG classification of BSF unigenes.

**Additional file 7: Figure S5.** Functional classification and pathway assignment of BSF unigenesby KEGG. The results are summarized in five main categories: A: Cellular Processes; B: Environmental Information Processing; C: Genetic Information Processing; D: Metabolism; E: Organismal Systems.

**Additional file 8: Table S3.** Differential expression unigenes.

**Additional file 9: Table S4.** GO annotation of differentially expressed unigenes.

**Additional file 10: Table S5.** KEGG annotation of differentially expressed unigenes.

**Additional file 11: Figure S6.** Kyoto Encyclopedia of Genes and Genomes (KEGG) analysis of genes involved in fatty-acid biosynthesis in BSF (the red box represents the presence of genesencoding enzyme).

**Additional file 12: Table S6.** The designed primers of the key enzymes involved in lipid metabolism for qRT-PCR.

**Additional file 13: Table S7.** Key enzymes related to fatty acid and triacylglycerol biosynthesis identified by annotation of the black soldier fly unigenes.

**Additional file 14: Table S8.** The nutritional composition of chicken feed.

## Data Availability

The raw data that are related to this work can be available upon requests from the correspondence author.

## Abbreviations

BSF: black soldier fly
BSFL: black soldier fly larvae
FA: fatty acid
1-d-L: one-day-old larvae
4-d-L: four-day-old larvae
8-d-L: eight-day-old larvae
12-d-L: twelve-day-old larvae
E-prepupa: early prepupa
L-prepupa: late prepupa
E-pupa: early pupa
L-pupa: late pupa
NCBI: National Center for Biotechnology Information
SRA: Short Read Archive
NR: NCBI non-redundant protein sequences
GO: Gene Ontology
KEGG: Kyoto Encyclopedia of Genes and Genomes
COG: Clusters of Orthologous Groups of proteins
NT: NCBI non-redundant nucleotide sequences
FPKM: fragments per kilobase per million
FDR: false discovery rate
LPL: lipoprotein lipase
CES1: carboxylesterase 1
UGT: glucuronosyltransferase
GLB1: beta-galactosidase
FAEE: fatty-acid ethyl ester
ER: endoplasmic reticulum
AKR1B: aldehyde reductase
ELOVL4: very long-chain fatty-acid protein 4
HSD17B4: (3R)-3-hydroxyacyl-CoA dehydrogenase/3a,7a,12a-trihydroxy-5b-cholest-24-enoyl-CoA hydratase/enoyl-CoA hydratase 2
DGK: diacylglycerol kinase (ATP)
ydfG: 3-hydroxy acid dehydrogenase/malonic semialdehyde reductase
ALDH: aldehyde dehydrogenase
HK: hexokinase
ADPGK: ADP-dependent glucokinase
GPI: glucose-6-phosphate isomerase
PFK: phosphofructokinase 1
FBP: fructose-1,6-bisphosphatase I
ALDO: fructose-bisphosphate aldolase, class I
GAPDH: glyceraldehyde 3-phosphate dehydrogenase
PGK: phosphoglycerate kinase
gpmA: 2,3-bisphosphoglycerate-dependent phosphoglycerate mutase
gpmI: 2,3-bisphosphoglycerate-independent phosphoglycerate mutase
PDC: pyruvate dehydrogenase complex
CS: citrate synthase
ACLY: ATP-citrate lyase
ACC: acetyl-CoA carboxylase
ACP: acyl-carrier-protein
FabD: *S*-malonyltransferase
BC: carboxylase
BCCP: biotin carboxyl carrier protein
CT: carboxyl transferase
KS: β-ketoacyl synthase
MAT: malonyl/acetyltransferase
DH: dehydrogenase
ER: enoylreductase
KR: β-ketoacylreductase
ACP: acyl carrier protein
ACSBG: long-chain-fatty-acid-CoA ligase
ACSL: long-chain acyl-CoA synthetase
ACS: acyl-coenzyme A synthetase
GPAT: glycerol-3-phosphate *O*-acyltransferase
DAG: diacylglycerol
PLPP: phosphatidate phosphatase
DGAT: diacylglycerol acyltransferase
ACAT: acyl-CoA:cholesterol acyltransferase
DGAT1: diacylglycerol *O*-acyltransferase 1
HZAU: Huazhong Agricultural University
