# Supplementary figures and images for: De novo transcriptome sequencing and analysis revealed the molecular basis of rapid fat accumulation by black soldier fly (Hermetia illucens, L.) for development of insectival biodiesel
Source: Biotechnol Biofuels. 2019 Aug 9;12:194. doi: 10.1186/s13068-019-1531-7 (PMC6688347; doi:10.1186/s13068-019-1531-7)

**Additional file 3: Figure S1** The length distribution of BSF unigenes

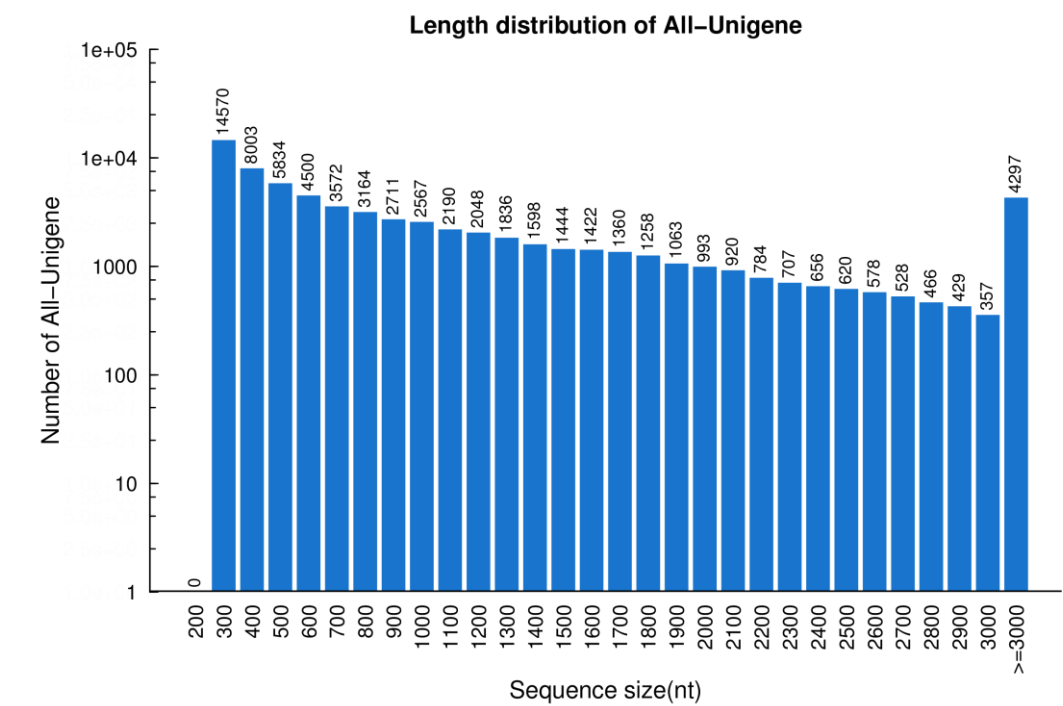

Supplement: Supplementary file 3 — Additional file 3: Figure S1. The length distribution of BSF unigenes. [file 13068_2019_1531_MOESM3_ESM.pdf]

**Additional file 6: Figure S4 COG classification of BSF unigenes.**

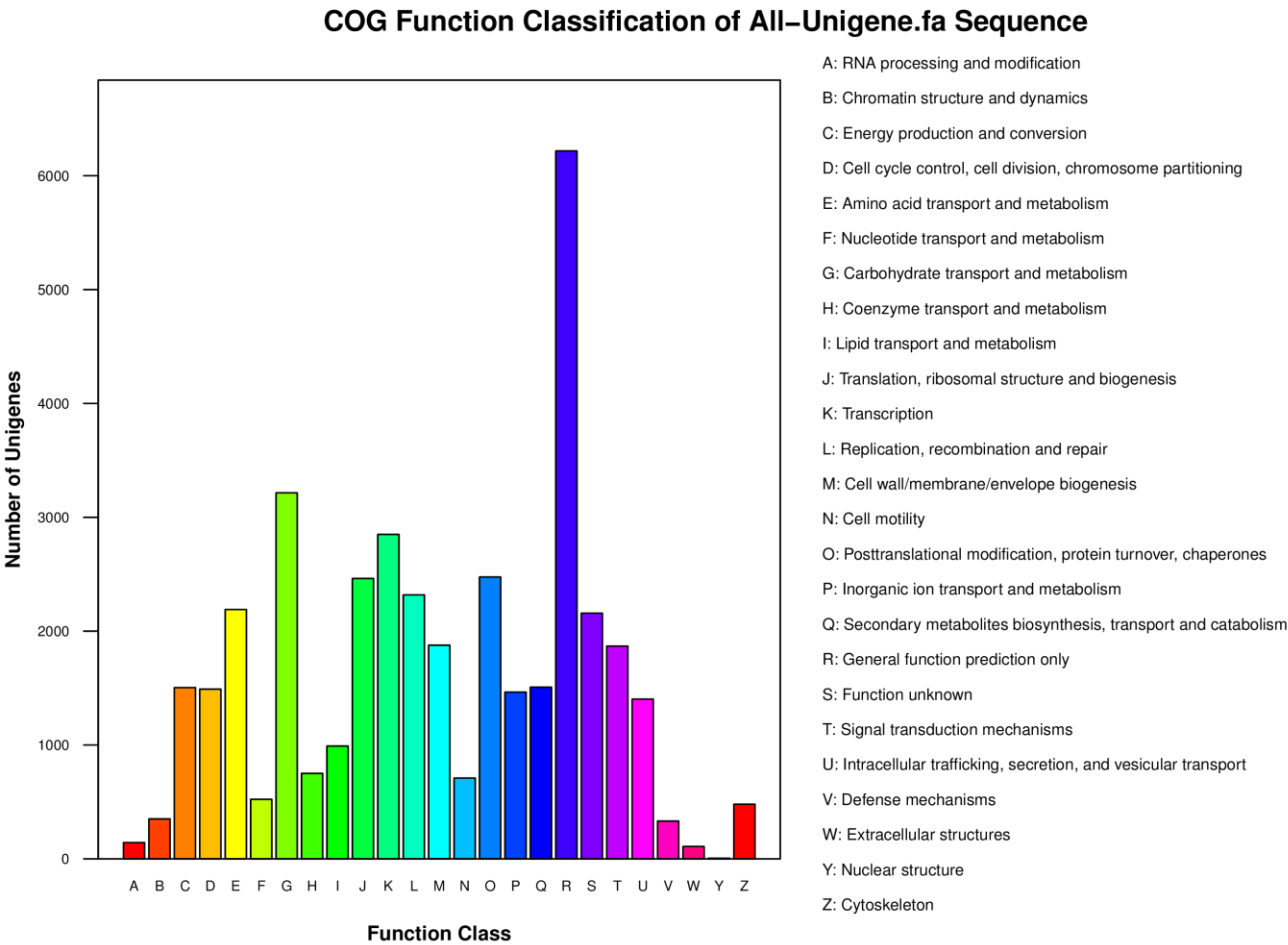

Supplement: Supplementary file 6 — Additional file 6: Figure S4. COG classification of BSF unigenes. [file 13068_2019_1531_MOESM6_ESM.pdf]
