# Supplementary material for: De novo transcriptome sequencing and analysis revealed the molecular basis of rapid fat accumulation by black soldier fly (Hermetia illucens, L.) for development of insectival biodiesel
Source: Biotechnol Biofuels. 2019 Aug 9;12:194. doi: 10.1186/s13068-019-1531-7 (PMC6688347; doi:10.1186/s13068-019-1531-7)

Additional file 4 Figure S2: Similarity analysis between BSF unigenes and NR database. **(A)** E-value (< $1e^{-5}$) distribution of top BLAST hits for each BSF unigene. **(B)** Similarity (> 17%) of BSF putative proteins with known proteins in NR database. **(C)** Top-hit species distribution of BLAST matches for BSF unigenes.

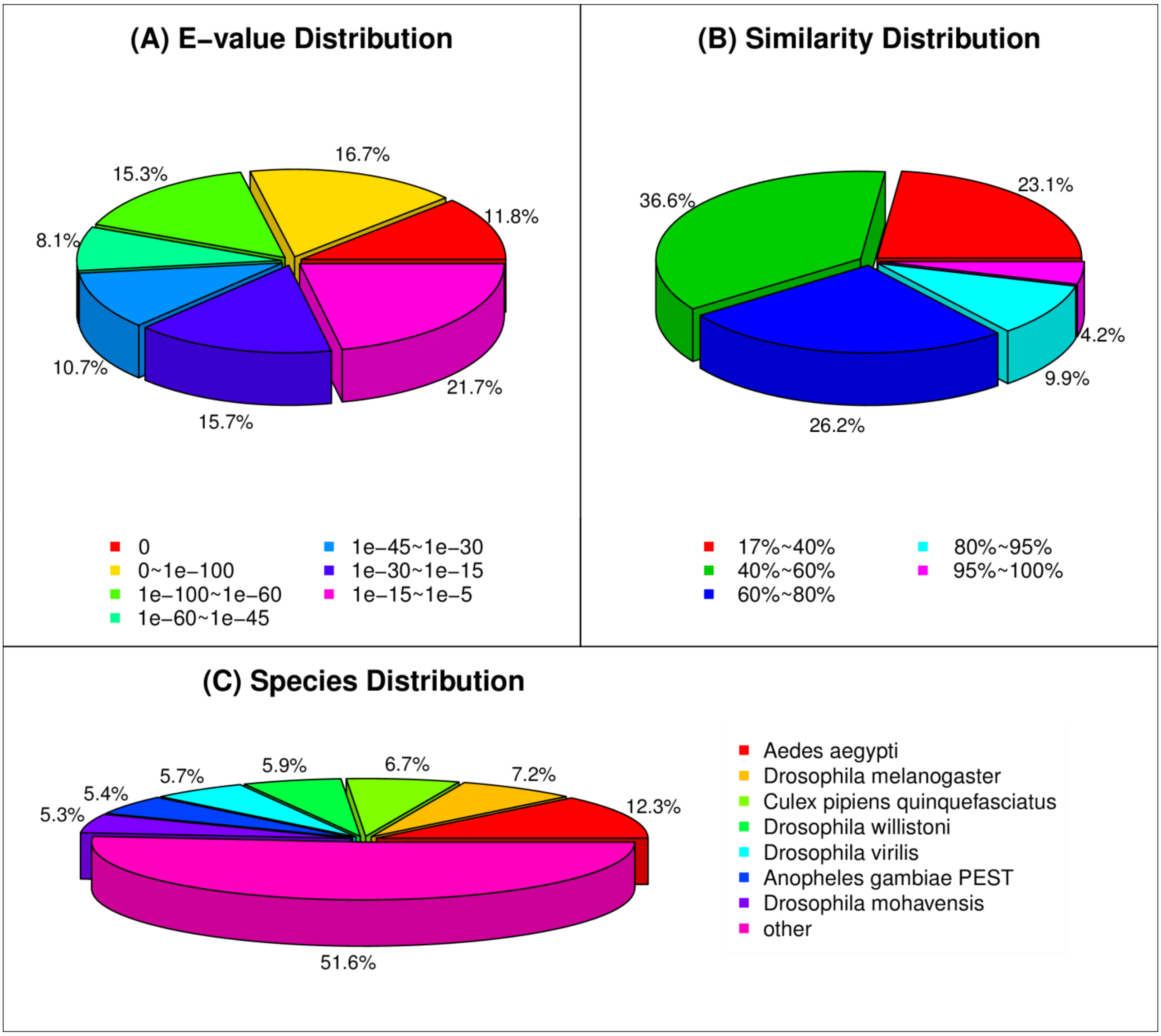

Supplement: Supplementary file 4 — Additional file 4: Figure S2. Similarity analysis between BSF unigenes and NR database. (A) e value (< 1e-5) distribution of top BLAST hits for each BSF unigene. (B) Similarity (> 17%) of BSF putative proteins with known proteins in NR database. (C) Top-hit species distribution of BLAST matches for BSF unigenes. [file 13068_2019_1531_MOESM4_ESM.pdf]
