# Supplementary material for: De novo transcriptome sequencing and analysis revealed the molecular basis of rapid fat accumulation by black soldier fly (Hermetia illucens, L.) for development of insectival biodiesel
Source: Biotechnol Biofuels. 2019 Aug 9;12:194. doi: 10.1186/s13068-019-1531-7 (PMC6688347; doi:10.1186/s13068-019-1531-7)

Additional file 5 Figure S3: Gene ontology (GO) classification of BSF unigenes. The left side and the right side of the panel show the percentage of genes and the number of genes that are classified to the three main categories, including biological process, cellular component, and molecular function.

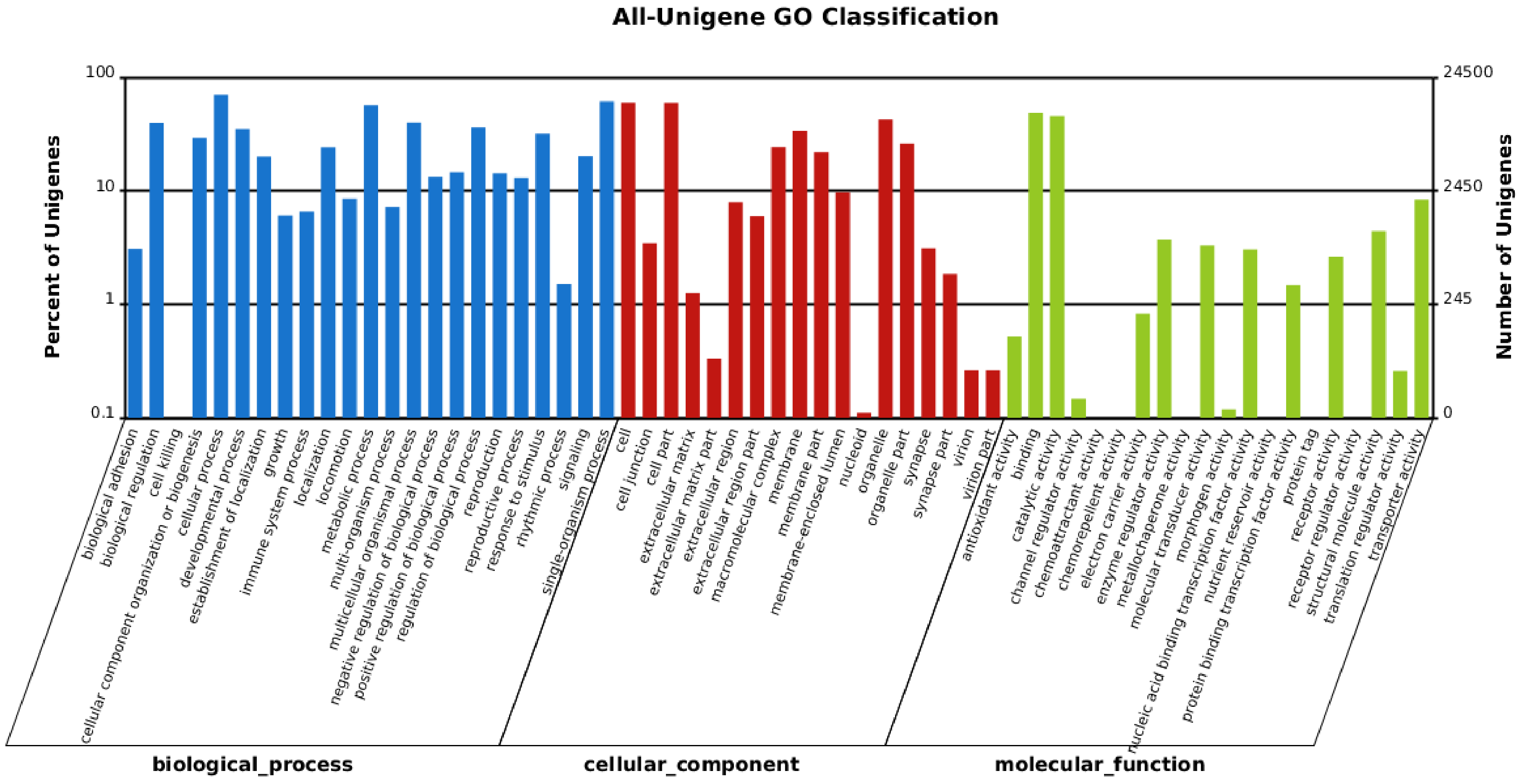

Supplement: Supplementary file 5 — Additional file 5: Figure S3. Gene ontology (GO) classification of BSF unigenes. The left side and the right side of the panel show the percentage of genes and the number of genes that are classified to the three main categories, including biological process, cellular component, and molecular function. [file 13068_2019_1531_MOESM5_ESM.pdf]
