# Supplementary material for: De novo transcriptome sequencing and analysis revealed the molecular basis of rapid fat accumulation by black soldier fly (Hermetia illucens, L.) for development of insectival biodiesel
Source: Biotechnol Biofuels. 2019 Aug 9;12:194. doi: 10.1186/s13068-019-1531-7 (PMC6688347; doi:10.1186/s13068-019-1531-7)

Additional file 7: Figure S5 Functional classification and pathway assignment of BSF unigenes by KEGG. The results are summarized in five main categories: A: Cellular Processes; B: Environmental Information Processing; C: Genetic Information Processing; D: Metabolism; E: Organismal Systems.

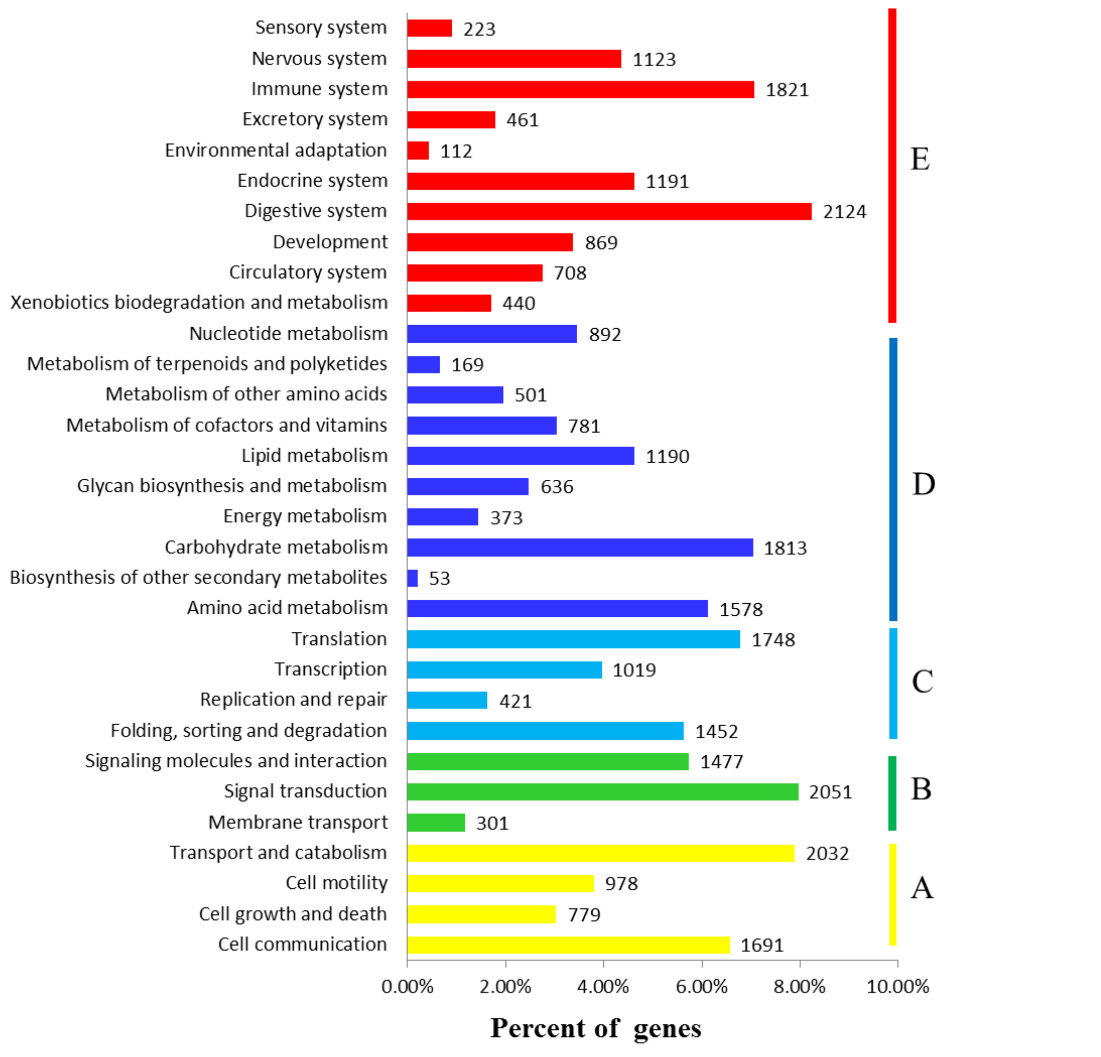

Supplement: Supplementary file 7 — Additional file 7: Figure S5. Functional classification and pathway assignment of BSF unigenesby KEGG. The results are summarized in five main categories: A: Cellular Processes; B: Environmental Information Processing; C: Genetic Information Processing; D: Metabolism; E: Organismal Systems. [file 13068_2019_1531_MOESM7_ESM.pdf]
