# Supplementary material for: De novo transcriptome sequencing and analysis revealed the molecular basis of rapid fat accumulation by black soldier fly (Hermetia illucens, L.) for development of insectival biodiesel
Source: Biotechnol Biofuels. 2019 Aug 9;12:194. doi: 10.1186/s13068-019-1531-7 (PMC6688347; doi:10.1186/s13068-019-1531-7)

Additional file 11 Figure S6 Kyoto Encyclopedia of Genes and Genomes (KEGG) analysis of genes involved in fatty acid biosynthesis in BSF (the red box represents the presence of genes encoding enzyme).

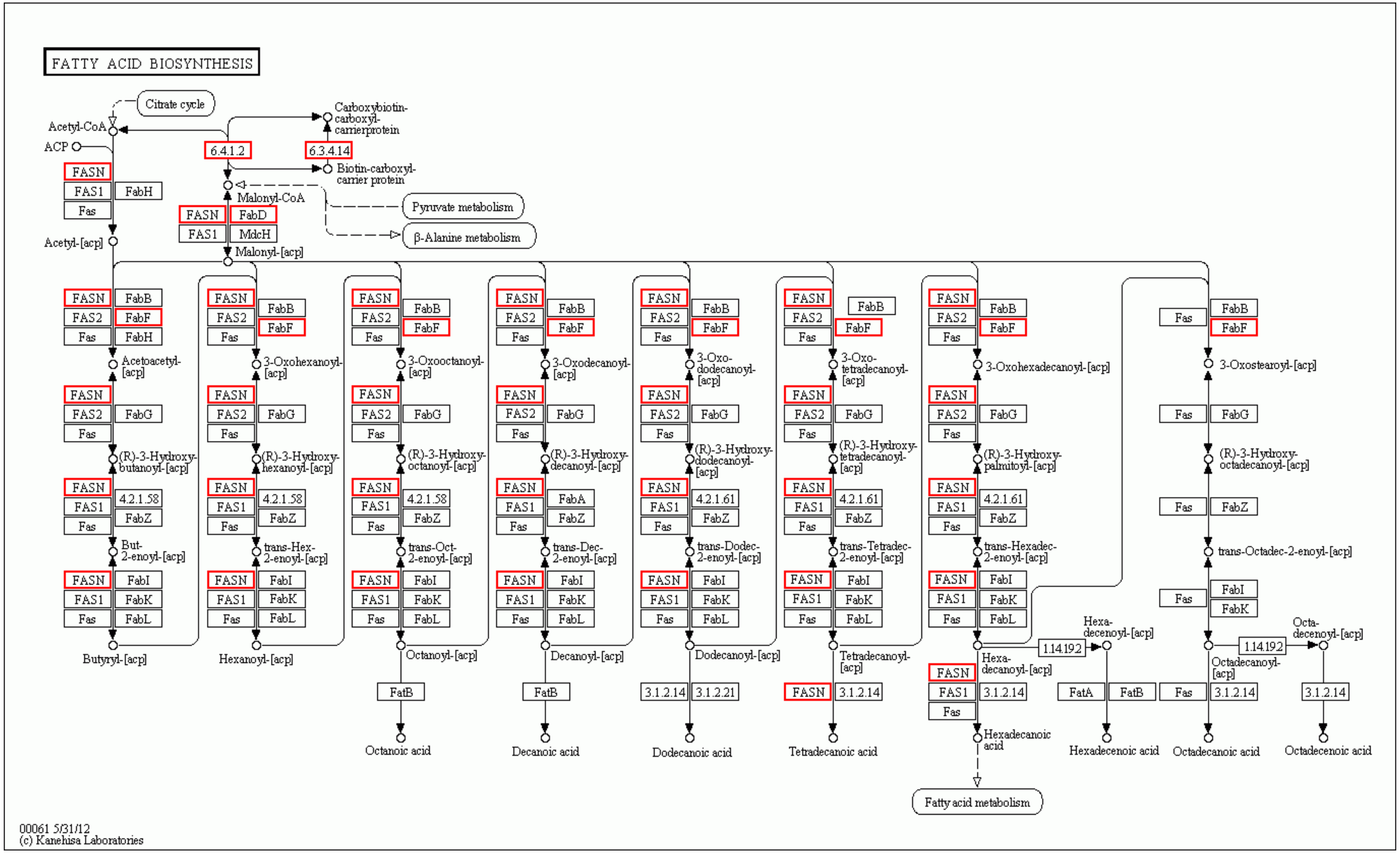

Supplement: Supplementary file 11 — Additional file 11: Figure S6. Kyoto Encyclopedia of Genes and Genomes (KEGG) analysis of genes involved in fatty-acid biosynthesis in BSF (the red box represents the presence of genesencoding enzyme). [file 13068_2019_1531_MOESM11_ESM.pdf]
